# Supplementary material for: ALDRESS: A Retrospective Pilot Study to Develop a Pharmacological Causality Algorithm for Drug Reaction with Eosinophilia and Systemic Symptoms (DRESS)
Source: J Clin Med. 2024 Apr 29;13(9):2622. doi: 10.3390/jcm13092622 (PMC11084416; doi:10.3390/jcm13092622)
Supplement: Supplementary file 1 [file jcm-13-02622-s001.zip › jcm-2969692-supplementary.pdf]

Supplementary *Online* Content

MATERIAL AND METHODS

The authors to give readers additional information about their work have provided this supplementary material.

The reference number corresponds to what is established in the original article

Table S1. RegiSCAR DRESS validation score [1s]

Parameter considered	The possible manifestations (for each parameter)		Point on RegiSCAR DRESS validation
FEVER ≥ 38.5 ºC	PRESENT		0
	ABSENT		-1
LYMP NODES	Lymphadenopathy (≥ 2 sites, > 1 cm)		+1
HEMATOLOGICAL CRITERIA	>5% atypical lymphocytes in peripheral smear		+1
	Absolute eosinophil count 700-1.499 cells/mm3		+1
	Absolute eosinophil count >1500 cells/mm3		+2
SKIN RASH	Maculopapular rash involving >50% of body surface area and does not satisfy features of rash suggestive of DRESS	Rash not showing 2/4 features suggestive of DRESS (edema, infiltration, purpura, scaling)	-1
		Generalized maculopapular rash	+1
	Maculopapular rash and two of the four features among facial edema, rash resolving with psoriasiform desquamation, infiltrated skin lesions and purpuric lesions on areas other than legs	Rash not showing 2/4 features suggestive of DRESS (edema, infiltration, purpura, scaling)	+1
		Generalized maculopapular rash	+1
INTERNAL ORGAN INVOLVEMENT	One internal organ involvement		+1
	Two or more internal organ involvement		+2
RESOLUTION	Resolution in > 15 days		-1
EXCLUDING OTHER CAUSES	Antinuclear antibody, blood culture, serology of HVA/HBV/HCV, Chlamydia/Mycoplasma. Core 1 if 3 tests on the following were performed and all were negatives: HAV, HBV, HCV, Mycoplasma, Chlamydia, ANA blood culture	None [+] and at least 3 [-]	+1

This classified suspected cases as definite (score 6 and above), probable (score 4 and 5), possible (score 2 and 3), and no DRESS (score <2)

Table S2. Algorithm for causality assessment of Drug Reaction with Eosinophilia and Systemic Symptoms (DRESS) syndrome, (ALDRESS)

1. Delay from initial drug component intake to onset of reaction (index day)*:

- Compatible (score +2): 1-12 weeks after initiation of treatment **
- Not fully supported (+1): between 3 -6 months
- No information (0)
- Incompatible chronology (–1): onset prior to drug initiation**/>6 months

2. Alternative: time to onset from cessation of the drug

- ≤ 15 days (except for slowly metabolized chemicals: > 15 days): +1

3. Drug notoriety:

- Known in the reference guidelines/literature (+2): recorded in the summary of product characteristics, or the adverse drug reaction was found in clinical trials, or the association has been found in cohort studies or in case-control studies.
- Occasionally known (+1): only found in published case reports.
- Unknown (0)
- Unrelated to the drug (–1): presence of confounding variables. Confounding variables appear when the estimate of a measure of association between the drug exposure and health status is distorted by the effect of ≥1 other variables that are also risk factors for the outcome of interest.

4. Evaluation of drug withdrawal:

- Improvement on withdrawal: (+2)
- Not withdrawing improves the effect (–2)
- Drug withdrawal does not improve effect (-2)
- No information (0)
- Death or irreversible effect (0)
- Not withdrawn, improves with specific (immunosuppression) treatment (0)

5. Risk factors:

- Previous collagen vascular diseases (ankylosing spondylitis, dermatomyositis, polyarteritis nodosa, psoriatic arthritis, rheumatoid arthritis, scleroderma, systemic lupus erythematosus) (+1)
- Chronic kidney disease for allopurinol (+1)
- Viral infection in the month prior to DRESS (+1)
- Genetic risk factor associated with DRESS by the drug*** (+1)

6. Rechallenge effect:

- Reappearance of the positive effect (+3)
- Negative, the effect does not reappear (–2)
- No re-exposure or no information (0)
- Death or irreversible effect (0)
- Positive for a different agent with the same active ingredient or parent drug according to ATC(+3)
- Positive for a different agent with confirmed cross reactivity to de culprit drug (+3)

7. Prechallenge

- Prior IV type hypersensitivity with this drug (+3)
- No re-exposure or no information (0)
- Positive prechallenge for a different agent with the same active ingredient or parent drug according to ATC (+3)
- Positive prechallenge for a different agent with the cross reactivity to de culprit drug (+3)

8. Concomitant drug administration:

- None or no information (0)
- Concomitant drug with incompatible time to onset (0)
- Concomitant drug with evidence for its role in this case as indicated by prospective studies (-1)
- Concomitant drug moderate evidence for its role in this case as indicated by prospective studies (-2)

- Concomitant drug with strong evidence for its role in this case as indicated by prospective studies (positive rechallenge or validated test) (-3)

9. **Microbiological evidence of herpes viral reactivation (typically VHH6/7/CMV/EBV in context of drug administration:** (+1)

10. **Inmunoalergy testing:**

- In vitro or in vivo validated test: positive for suspicious/parent drug or active ingredient or metabolites (+3)
- In vitro or in vivo validated test: Negative (0) or not performed (0)

11. **Alternative cause****:**

- If onset can be justified by other clinical entity (-3).
- If onset cannot be justified by other clinical entity (0).

SCORE: 0-2 Highly unlikely; 3-4 possible; probable: 5-6; highly probable 7-8; > 9 defined causation.

(*) Index day is considered to be the day on which prodromal symptoms / signs occurred, or in their absence, the day of acute rash. (**) Except for NSAIDs and for antibiotics between 3 and 7 days[Um et al. J Investig Allergol Clin Immunol 2010; Vol. 20(7): 556-562 (***) Genetic risk factors are available: The HLA Adverse Drug Reaction Database available at http://www.allelefrequencies.net/hla-adr/default.asp; PharmaGKB Database available at https://www.pharmgkb.org/. (****)there are a number of differential diagnostic considerations, because the clinical manifestations of DRESS can mimic other diseases such as viral primoinfections: they include EBV or CMV-induced infectious mononucleosis (IM), parvovirus B19 infection, measles infection, dengue virus infection, Coxsackie virus infection, Kawasaki disease, and Kikuchi-Fujimoto disease, whose patients may present with fever, skin rashes, lymphadenopathy, and internal organ involvement.
Abbreviations: ATC, anatomical therapeutic chemical. Similar Drug= same ATC code up to the fourth level (chemical subgroups).

Table S3. Algorithm of the Spanish Pharmacovigilance System (ASPS) [2s]

Algorithm of the Spanish Pharmacovigilance System
**1. The chronology referred to as the interval between drug administration and effect :**
1. Compatible (score +2)
2. Not totally compatible (+1)
3. No information (0)
4. Incompatible chronology (-1)
5. Particular case of syndrome of abstinence (+2).
**2. The literature, defining the degree of knowledge of the relationship between the drug and the effect:**
1. Known in the literature of reference (+2): collected in Summary of Product Characteristics or in books of reference (Martindale, Meyer's) .
2. Occasionally known (+1): only found in published cases reports.
3. Unknown (0)
4. There is pharmacological information against relationship between medicine and the adverse effect (-1)
3. **The evaluation of drug withdrawal:**
1. Improves with the withdrawal (+2)
2. Does not improve with withdrawal (-2)
3. No improvement and no withdrawal (+1)
4. Improves and there is no withdrawal (-2)
5. No information (0)
6. Death or irreversible effect (0)
7. Improves by development of tolerance, to despite not to withdraw (+1)
8. Improves with symptomatic treatment to despite not to withdraw (+1)
**4. The rechallenge effect:**
1. Positive effect reappearance (+3)
2. Negative, the effect does not reappear (-1)
3. No re-exposure or no information (0)
4. Death or irreversible effect (0)
5. Positive for a different specialty with the same active ingredient or parent drug (+1)
**5. Alternative causes:**
1. Yes, an illness or other drugs (-3).
2. Similar likelihood for drug and other causes (-1)
3. Missing information (0)
4. There is not any alternative cause (+1)
**6. Contributing factors:**
1. Yes (+1).
2. No (0)
**7. Complementary explorations:**
1. Yes (+1).
2. No (0)
**Categories according to final SCORE:**<br>• Not classified (lack of data)/Improbable: <0<br>• Conditional: 1-3<br>• Possible: 4-5<br>• Probable: 6-7<br>• Defined: ≥ 8

References

Ref 1s. Kardaun SH, Sidoroff A, Valeyrie-Allanore L, Halevy S, Davidovici BB, Mockenhaupt M, Roujeau JC. Variability in the clinical pattern of cutaneous side-effects of drugs with systemic symptoms: does a DRESS syndrome really exist? Br J Dermatol. 2007 Mar;156(3):609-11. doi: 10.1111/j.1365-2133.2006.07704.x.

Ref 2s. Aguirre C, García M. Evaluación de la causalidad en las comunicaciones de reacciones adversas a medicamentos. Algoritmo del Sistema spañol de Farmacovigilancia [Causality assessment in reports on adverse drug reactions. Algorithm of Spanish pharmacovigilance system]. Med Clin (Barc). 2016 Nov 18;147(10):461-464. Spanish. doi: 10.1016/j.medcli.2016.06.012. Epub 2016 Jul 20.
